# Mitral Papillary Fibroelastoma Revealed by Central Retinal Artery Occlusion

**DOI:** 10.7759/cureus.99033

**Published:** 2025-12-12

**Authors:** Zineb Rahali, Badre El Boussaadani, Zainab Raissouni

**Affiliations:** 1 Cardiology, Mohammed VI University Hospital Center, Abdelmalek Essadi University, Tangier, MAR

**Keywords:** cardiac tumor, embolic event, mitral valve, papillary fibroelastoma, surgical excision, transesophageal echocardiography, transthoracic echocardiography

## Abstract

Papillary fibroelastoma (PFE) is a very rare benign primary cardiac tumor that preferentially affects heart valves and may lead to severe embolic events. Its diagnosis is now easily suggested by echocardiography, and surgical excision is a safe and effective treatment to prevent recurrence.

We report the case of a patient referred for the etiological evaluation of a central retinal artery occlusion, in whom transthoracic and transesophageal echocardiography (TEE) revealed a fibroelastoma located on the mitral valve. This case highlights the importance of a comprehensive cardiac evaluation in any unexplained embolic event, even when the initial presentation is extra-cardiac.

## Introduction

Papillary fibroelastoma (PFE) is a rare, benign primary cardiac tumor and the most common neoplasm of the cardiac valves [1-3]. Although histologically benign, PFEs carry a significant embolic risk due to their high mobility and delicate papillary structure, and they are increasingly identified incidentally with modern imaging techniques [4-6]. Clinical manifestations range from silent forms to severe embolic events, including stroke, retinal artery occlusion, myocardial infarction, or peripheral ischemia.

A key diagnostic challenge is distinguishing PFEs from other intracardiac masses, particularly infective vegetations, thrombi, and myxomas, as treatment implications differ substantially [7-9]. Vegetations are typically associated with fever, elevated inflammatory markers, and positive blood cultures; thrombi usually occur in the setting of atrial fibrillation or left ventricular dysfunction; and myxomas are generally larger, less mobile, and attached by a broad stalk [10-12].

Echocardiography is the cornerstone of evaluation. Transesophageal echocardiography (TEE) can strongly suggest the diagnosis based on characteristic morphology, but it cannot confirm it; definitive diagnosis requires histopathological examination [13]. When diagnostic uncertainty remains, cardiac CT and MRI provide additional anatomical and tissue-characterization details to refine the differential diagnosis [14-16].

We report a case of mitral valve PFE revealed by central retinal artery occlusion, emphasizing the importance of considering cardiac sources in the setting of unexplained embolic events.

## Case presentation

A 66-year-old woman with no significant cardiovascular history presented with sudden, painless loss of vision in her left eye. Ophthalmologic evaluation confirmed a central retinal artery occlusion. She was referred for evaluation of a possible embolic etiology.

On examination, the patient was afebrile, hemodynamically stable, and had no cardiac murmur, peripheral stigmata of infective endocarditis, or signs of heart failure. Laboratory tests showed normal white blood cell count, C-reactive protein, and erythrocyte sedimentation rate. Three sets of blood cultures were negative. These findings, combined with the absence of risk factors or clinical features of infection, made infective endocarditis unlikely.

Electrocardiography demonstrated sinus rhythm without conduction abnormalities, and 24-hour Holter monitoring did not reveal atrial fibrillation or other arrhythmias. The patient had no history of thromboembolic disease, hypercoagulability, or left ventricular dysfunction. Carotid Doppler ultrasound was normal, and brain imaging did not reveal cerebral ischemia.

**Figure 1:**
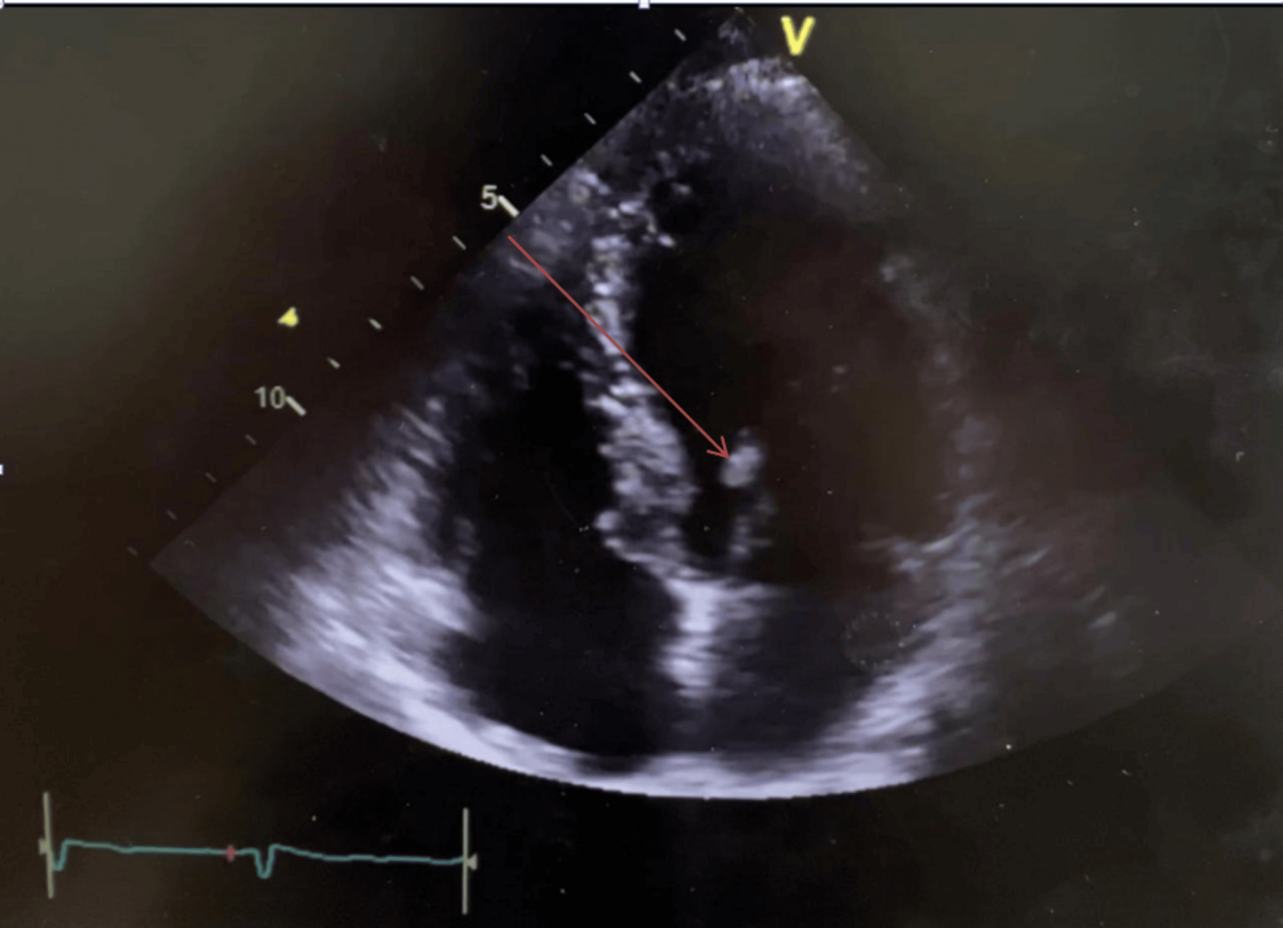
Apical four-chamber view showing an echogenic mass attached to the anterior mitral leaflet, oscillating within the left ventricle (arrow).

TTE identified a small, highly mobile, pedunculated echogenic mass attached to the ventricular side of the anterior mitral leaflet (Figure 1).

**Figure 2:**
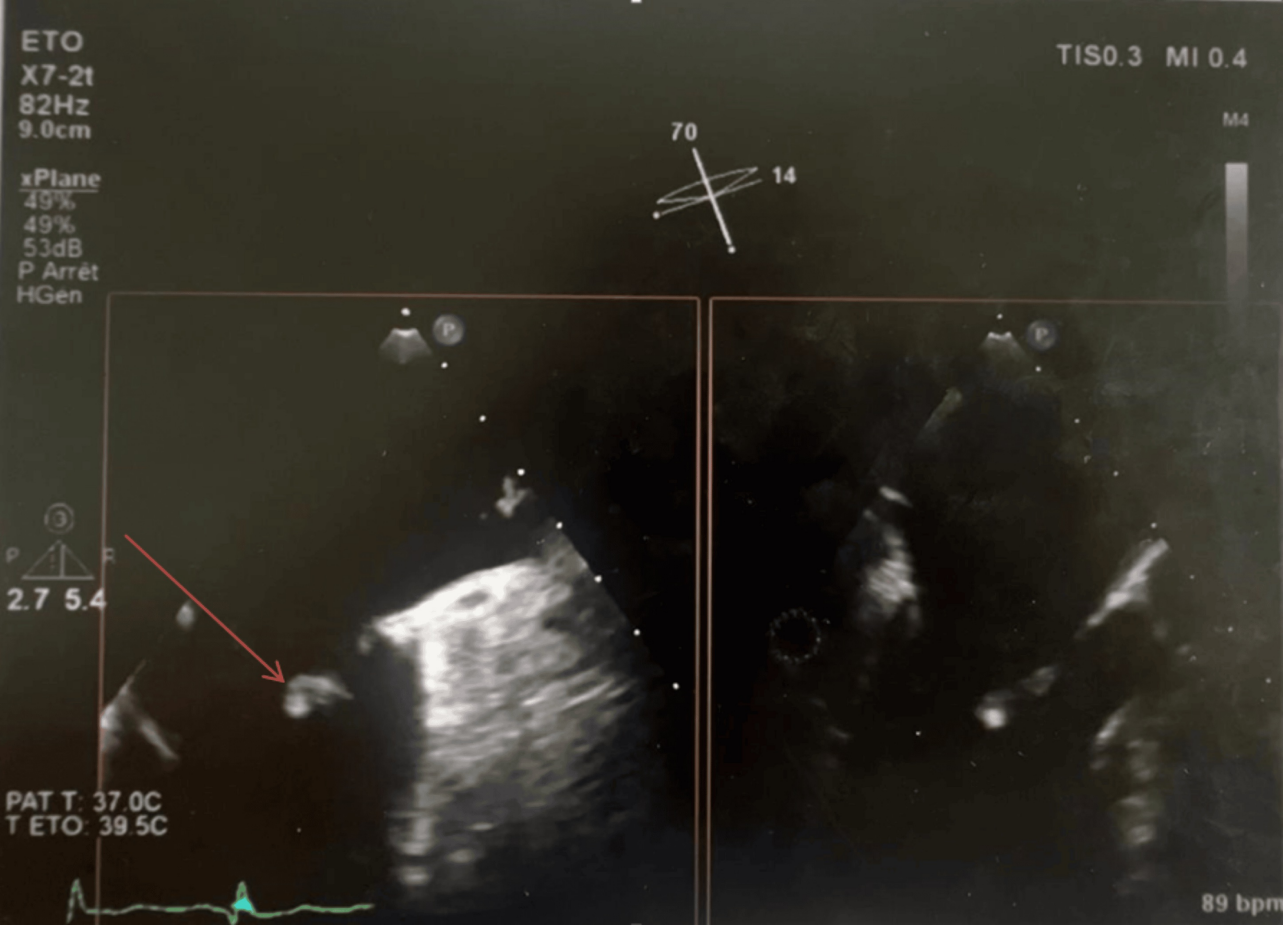
Two-dimensional transesophageal echocardiography showing, in two views, a mobile pedunculated mass attached to the anterior mitral leaflet (arrow).

TEE provided better characterization, revealing a frond-like lesion with independent motion, strongly suggestive of PFE; however, we emphasize that echocardiography can only raise suspicion, as definitive diagnosis requires histopathological confirmation (Figure 2).

**Figure 3:**
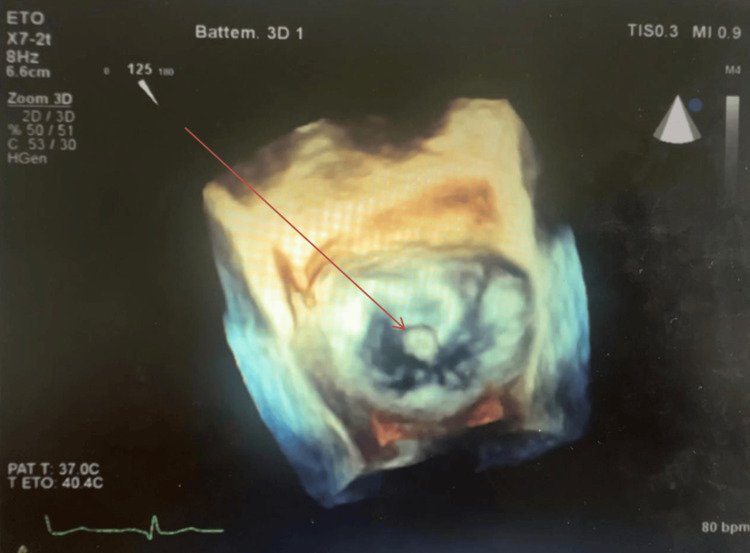
Three-dimensional echocardiography showing the location of the mass on segment A2 of the mitral valve (arrow).

Three-dimensional TEE further localized the mass to segment A2 of the anterior mitral leaflet (Figure 3). No valvular dysfunction was observed.

Given the documented embolic event and the high embolic potential associated with such mobile valvular masses, surgical excision was recommended. The patient underwent complete resection of the mass with preservation of the mitral valve. Histopathological examination confirmed the diagnosis of PFE. The postoperative course was uneventful, and cardiology and ophthalmology follow-up were arranged.

## Discussion

PFE is a rare benign cardiac tumor belonging to the spectrum of primary cardiac neoplasms, which themselves have a low overall incidence [1]. Among valvular tumors, PFEs are considered the most frequent, although their exact prevalence remains uncertain [2]. They originate predominantly from the valvular endocardium, most commonly involving the aortic and mitral valves, and typically occur in older adults without significant sex predilection. Their usual location, on the downstream side of the aortic valve or the ventricular surface of the mitral valve, is an important distinguishing feature from infective endocarditis, which more often affects the upstream surface exposed to turbulent flow.

Clinically, PFEs are frequently asymptomatic and may be discovered incidentally. Their importance lies in their well-recognized potential to cause systemic embolic events, including neurological complications, coronary embolism, or peripheral arterial occlusion [3]. Left-sided PFEs are particularly emboligenic because of their high mobility and frond-like papillary architecture, which favors tumor fragmentation or thrombus detachment [4,5]. Retinal artery occlusion, as seen in our patient, is a rare but documented manifestation of this embolic potential.

Echocardiography remains the cornerstone of diagnosis. Transthoracic echocardiography typically identifies a small, mobile, pedunculated mass, while TEE, although it cannot confirm the diagnosis, provides superior spatial resolution and allows a more precise evaluation of tumor mobility and attachment [6,7]. Reported sensitivities reach approximately 62% for transthoracic echocardiography and 77% for TEE. When echocardiographic findings are incomplete or additional anatomical detail is needed, cardiac CT and MRI are valuable complementary methods to help differentiate PFEs from other masses such as thrombi or vegetations [8,9].

Differential diagnosis is essential, particularly to distinguish PFE from conditions with very different management strategies. Infective endocarditis vegetations usually present with fever, elevated inflammatory markers, positive blood cultures, and regurgitant valvular lesions. They typically arise on the upstream surface of the valve. In our case, the patient had no fever, no biological inflammatory syndrome, negative blood cultures, and no valvular destruction, making vegetation unlikely. Intracardiac thrombus generally occurs in patients with atrial fibrillation, left ventricular dysfunction, or hypercoagulable states. Our patient remained in sinus rhythm, had normal ventricular function, and had no thrombotic risk factors, reducing the likelihood of thrombus. Other cardiac tumors, such as myxomas, usually arise from non-valvular sites (for example, the interatrial septum) and have different morphological characteristics.

Definitive diagnosis relies on histopathological examination. PFEs are typically non-vascularized lesions composed of a central collagen-rich core covered by elastic fibers, connective tissue, and an endothelial layer [10]. Macroscopically, they present as small pedunculated masses with multiple papillary projections, giving them a characteristic sea-anemone appearance when placed in saline [11]. Cytogenetic abnormalities have also been described, although their clinical significance remains uncertain [12].

Given the high embolic potential of PFEs, particularly when they are mobile and left-sided, surgical excision is widely recommended [13]. Surgery is usually curative, with low operative risk and excellent long-term outcomes [14]. Medical therapy alone, including antiplatelet or anticoagulation treatment, is generally insufficient to prevent recurrent embolic events and is reserved for non-surgical candidates [15,16].

In our patient, the initial presentation with central retinal artery occlusion and the echocardiographic identification of a mobile mass on the ventricular side of the mitral valve were highly suggestive of PFE rather than a thrombus or vegetation. Surgical resection allowed definitive diagnosis and prevented further embolic complications.

## Conclusions

PFE are the most common primary valvular cardiac tumors and carry a significant embolic risk, particularly when mobile and located on the left side of the heart. Their typical attachment on the downstream surface of the aortic valve or the ventricular aspect of the mitral valve helps distinguish them from infective endocarditis vegetations. Echocardiography, while unable to provide a definitive diagnosis, plays a central role in raising suspicion, with CT and MRI serving as valuable complementary tools when differentiation from thrombus or vegetation remains uncertain.

This case highlights that even rare cardiac tumors may present with isolated extracardiac embolic manifestations such as central retinal artery occlusion. Early recognition and timely surgical excision remain essential to prevent recurrent embolic events and ensure an excellent long-term prognosis.
